# Episensitization: Defying Time’s Arrow

**DOI:** 10.3389/fonc.2015.00134

**Published:** 2015-06-11

**Authors:** Bryan T. Oronsky, Arnold L. Oronsky, Michelle Lybeck, Neil C. Oronsky, Jan J. Scicinski, Corey Carter, Regina M. Day, Jose F. Rodriguez Orengo, Maribel Rodriguez-Torres, Gary F. Fanger, Tony R. Reid

**Affiliations:** ^1^EpicentRx, Inc., Mountain View, CA, USA; ^2^InterWest Partners, Menlo Park, CA, USA; ^3^CFLS LLC, San Jose, CA, USA; ^4^Walter Reed National Military Medical Center, National Cancer Institute, Bethesda, MD, USA; ^5^Uniformed Services University of the Health Sciences, Bethesda, MD, USA; ^6^Fundación de Investigación, Rio Piedras, PR, USA; ^7^Moores Cancer Center, University of California San Diego, La Jolla, CA, USA

**Keywords:** episensitization, epigenetics, epigenomic, resensitization, RRx-001, oncology

## Abstract

The development of cancer is driven by complex genetic and epigenetic changes that result in aberrant and uncontrolled cellular growth. Epigenetic changes, in particular, are implicated in the silencing or activation of key genes that control cellular growth and apoptosis and contribute to transformative potential. The purpose of this review is to define and assess the treatment strategy of “episensitization,” or the ability to sensitize cancer cells to subsequent therapy by resetting the epigenetic infrastructure of the tumor. One important facet is resensitization by epigenetic mechanisms, which goes against the norm, i.e., challenges the long-held doctrine in oncology that the reuse of previously tried and failed therapies is a clinically pointless endeavor. Thus, episensitization is a hybrid term, which covers recent clinically relevant observations and refers to the epigenomic mechanism of resensitization. Among the many formidable challenges in the treatment of cancer, the most inevitable is the development of acquired therapeutic resistance. Here, we present the basic principles behind episensitization and highlight the evidence suggesting that epigenetically mediated histone hypoacetylation and DNA hypermethylation events may reverse clinical drug resistance. The potential reversibility of epigenetic changes and the microenvironmental impact of epigenetic control on gene expression may mediate a return to a baseline state of treatment susceptibility. Episensitization is a novel and highly practical management strategy both to prevent the practice of permanent treatment discontinuation with the occurrence of resistance, which rapidly exhausts remaining options in the pharmaceutical armamentarium and to significantly extend patient survival. Accordingly, this review highlights several epigenetic agents including decitabine, vorinostat, entinostat, 5-azacitidine, oncolytic viruses, and RRx-001.

## Introduction

The purpose of this review is to define and critically appraise the treatment strategy of “episensitization” or resensitization by epigenetic means, which challenges the long-standing, largely implicit tradition in oncology that the reuse of previously tried therapies constitutes a risky business with little real benefit to the patient. Unsurprisingly, given this implicit stigma, the literature about resensitization is limited. Nevertheless, over the years, several sources have provided a somewhat vague definition of resensitization^1^, with a lack of appositive context clues or morphemic analysis, as “A second or subsequent sensitization, especially following desensitization.” In an attempt to avoid confusion, we define resensitization as follows: “the renewal of clinical benefit from chemotherapy, immunotherapy, or radiotherapy that was previously effective but to which the tumor had become desensitized or resistant.” This definition has several important and potentially controversial consequences. One is that episensitization has the potential to reverse the direction of molecular entropy (since the micro-morphological and molecular landscape of cancer is defined by maximum entropy) and thereby defy time’s arrow. Episensitization is a neologism, coined by Oronsky, Scicinski, Fanger, and Reid, which covers recent clinically relevant observations and refers to the epigenomic mechanism for resensitization (1).

The complexity of cancer, which includes tumor heterogeneity, the lack of specific actionable targets, a mercurial microenvironment, and immunosuppression, makes it an extremely challenging disease to treat; and any clinical benefit from treatment is often tempered by the inevitable development of acquired therapeutic resistance and tumor progression (2, 3). Progression is a particularly apt term to describe the discrete, forward marching sequence of treatment steps as resistance develops and the disease worsens over time. The apparent reversibility of resistance by episensitization has the potential to run the arrow of time backwards, replacing progression with regression. This one-way arrow of temporality defines entropy, a thermodynamic expression of increasing disorder over time that has been repeatedly applied to and closely associated with the features of cancer tissue or cells.

According to the Second Law of Thermodynamics, natural processes tend to move from ordered to disordered states. For example, the dissolution of sugar cubes in coffee or the dispersion of perfume molecules in the air is irreversible processes, which from experience never proceed in the opposite direction: no matter how long the interval, neither the sugar nor the perfume molecules will spontaneously reverse their trajectories and reform as cubes or reassemble in the bottle, respectively. In other words, what is done cannot be undone, time moves in one direction and one direction only, forward not backwards, irreversibly.

Similarly, over time and after treatment, cancer cells tend to become more resistant to therapy. In this way, the one-way arrow of temporality points toward inevitable treatment failure as cancer patients traverse multiple lines of therapy in a more or less linear sequence, first-line, second-line, third-line etc., until the only remaining option is hospice or a clinical trial. This review presents the clinical evidence for episensitization, i.e., the reversal of drug resistance with the restitution of clinical activity in terms of epigenetic gene reactivation mediated by histone hypoacetylation and DNA hypermethylation. Accordingly, clinical trials with several potential reset-button-pushing epigenetic agents are highlighted in this review. These epigenetic agents are decitabine (DAC), 5-azacitidine (AZA), entinostat, resminostat, and RRx-001.

Worldwide, cancer is a leading cause of mortality (4) and resistance to therapy, whether *de novo* or acquired, is a chief culprit. Episensitization is a highly practical management strategy both as an antidote to the practice of permanent discontinuation of treatments at each line of therapy with the occurrence of resistance, which rapidly exhausts remaining options in the pharmaceutical armamentarium and as a blueprint to significantly extend patient survival.

## Keywords Defined

“Epigenetics” possesses several context-dependent definitions (5). As the “next big thing” in oncology, epigenetics is an eminently flexible concept that accommodates a range of meanings including phenotypic plasticity and adaptive capacity. However, in the context of this review, epigenetics refers to reversible changes in gene expression, requiring active maintenance (6) (unlike genetic modifications) and potentially manipulatable by small molecule DNA methyltransferase (DNMT) and histone deacetylase (HDAC) inhibitors. Epigenetic perturbations lead to alterations in gene expression, which may drive malignant transformation and contribute to the development of resistance to anti-cancer therapies (7). According to Brown et al., “the high rate of epigenetic change in tumors generates diversity in gene expression patterns that can rapidly evolve through drug selection during treatment, leading to the development of acquired resistance” (8).

For the purposes of this review, “resensitization” refers to clinically meaningful benefit in the context of previous exposure to a previously effective but now refractory medication while “sensitization” indicates improved clinical benefit in the absence of previous drug exposure. Episensitization is a blend word of epigenetics and sensitization coined by Oronsky, Scicinski, Fanger, and Reid that refers to the reversal of epigenetic changes associated with resistance to treatment (9). An episensitization-related keyword is “priming,” which refers to increased drug sensitivity, presumably due to altered gene expression. In this context, epigenetic agents “prime the pump” or reprogram the tumor so that it is poised to respond to further treatment. A “primed” state may be short-lived or longer-term (see the multi-epigenetic agent, RRx-001). Epi-resensitization is a sub-category under the general rubric of episensitization.

This review serves as an introduction to the relatively under-explored strategy of tumor resensitization. Definitions, implications, and future directions are discussed.

## Cancer Ecology

Cancer has been described (10) as an ecological process whereby tumor cells reengineer and reorganize their environment, imposing significant physiologic stress (11), in order to effectively out-compete the indigenous populations of normal cells in specific biological niches. The tumor effects a “chaotic” rearrangement (12) that is characterized by specialized adverse hallmark traits, e.g., hypoxia, nutrient deprivation, poor blood flow, low pH, and increased oxidative stress and provides a favorable microenvironment for tumor cells relative to normal tissue. Further, effective anti-cancer therapies may accentuate oncogenic epigenetic reorganization, paradoxically leading to the increased development of aggressive, drug-resistant tumor phenotypes.

The principle of “ecogenetic feedback” in evolutionary biology (13) describes a synergistic interplay between ecological and genetic effects. In a similar way, cancer cells dynamically regulate transcription to match phenotype with the prevailing microenvironment. Thus, tumor cells epigenetically upregulate and downregulate the expression of particular genes (14) in real-time, resulting in reprograming-associated epigenetic/transcriptional signatures (15). The collective behavior of these specific interactions of gene and protein expression patterns is to optimize fitness of the tumor cells in particular microenvironmental conditions. This adaptive flexible responsiveness is known as phenotypic plasticity (16) and greatly increases the competitiveness of the cancer cell relative to non-neoplastic cells, allowing the tumor to adjust its phenotype to the local microenvironment, provided that the costs of an adaptive plastic response are not selectively prohibitive, since the process of protein synthesis is energetically expensive (17). An example of this is the repression of oxidative metabolism that occurs in the presence of aerobic glycolysis (the Warburg effect) (18), a metabolic hallmark of cancer cells, whereby requisite ATP (adenosine triphosphate) is produced via increased glycolytic flux. Cancer cells adapt to carefully adhere to an energy budget – glycolysis is an inefficient process for ATP production, the net yield of ATP per molecule of glucose degraded under anaerobic conditions, 2 ATP, is far inferior to complete oxidation of one glucose molecule by oxidative phosphorylation, which generates up to 36–38 ATP molecules (19); hence, excess capacity is not unlimited.

Treatment of cancer with molecularly targeted agents that modulate specific genetic abnormalities like VEGF (vascular endothelial growth factor), EGFR (epidermal growth factor receptor), or BRAF has met with limited success because their specificity is too narrow: the built-in phenotypic plasticity or flexibility (20, 21) of the cancer epigenome facilitates rapid and recurrent compensatory adaptation, and the energetic costs that may constrain adaptation are evidently not prohibitively expensive. Despite initial benefit of the treatment, tumor progression inevitably ensues (22), due to the emergence of resistance through altered drug metabolism, active drug export, modified expression or function of the drug target, or through the activation of a downstream molecule, all of which are risk factors for aggressive tumor behavior and shortened overall patient survival.

Like molecularly targeted agents, epigenetic compounds “act” locally and selectively on specific enzymes. But, unlike molecularly targeted agents, epigenetic compounds “behave” globally, pleiotropically affecting the expression of *multiple* genes, which disrupts the complex interplay between multiple interacting and interdependent cellular and microenvironmental elements, consequently reducing tumor fitness. Through a relatively small perturbation, the inhibition of epigenetic modulators like HDACs or DNMT, the pattern of coordinated gene expression in the tumor is altered, resulting in a multiplier effect (23). For example, while inhibition of HDAC activity affects the expression of only 5–20% of protein-coding genes (24), the fact that these proteins converge on pathways governing gene expression, cell proliferation, cell migration, cell death, immune pathways, and angiogenesis creates a sum of sudden temporal changes in the microenvironment, forcing the tumor to counter by expending energy (expend to defend) and tipping the balance in favor of chemosensitivity (25).

## Drug-Resistant Traits are Energetically Expensive

Dr. Robert Gatenby, a practitioner of mathematical medicine, characterizes the induction of resistance to cytotoxicity as an energy intensive process, requiring ATP expenditure to upregulate xenobiotic metabolism, DNA repair, and inactivation of cell death pathways (26). He postulates that treatment failure in oncology is, for the most part, an iatrogenic phenomenon, caused by overtreatment. In other words, the standard supra-inhibitory, maximally tolerated dose (MTD) model, paradoxically facilitates the survival of resistant clones.

It is well established that tumors are heterogeneous (27). Within that heterogeneity, drug-intolerant cells are outcompeted by drug-sensitive variants for resources within the tissue microenvironment. The acquisition of a refractory phenotype is energetically expensive and inefficient (28). However, under the selection pressure of MTD chemo- or radiotherapy (29), resistant clones opportunistically replace the sensitive cells eradicated during treatment. By contrast, if chemosensitive cells are not displaced, they impair the colonization and dissemination of resistant populations. An analogous situation occurs in particular body niches – skin, vagina, GU (genitourinary), and GI (gastrointestinal) tracts – where the microbial flora normally limits the adherence of exogenous pathogens or indigenous opportunistic commensals (30) unless antibiotic administration perturbs the ecological balance.

For this reason, Gatenby proposes a non-MTD paradigm called adaptive therapy (28), which preserves the viability and the primacy of the drug-sensitive cells through continuous adjustment of lower dose chemo over less frequent intervals to maintain a mathematically defined target tumor burden. Proof-of-principle studies have been completed in tumor bearing mice but no human data are available. Similar sensitization/resensitization strategies, which attempt to achieve a stable population of chemotherapy-sensitive cells that suppress the growth of resistant populations, include treatment holidays, metronomic dosing, OPTIMOX (31, 32), and COIN-like (33) intermittent treatment, and hypomethylating agents.

## The Epigenetic Landscape and Retreatment After Drug Holidays

**Figure 1:**
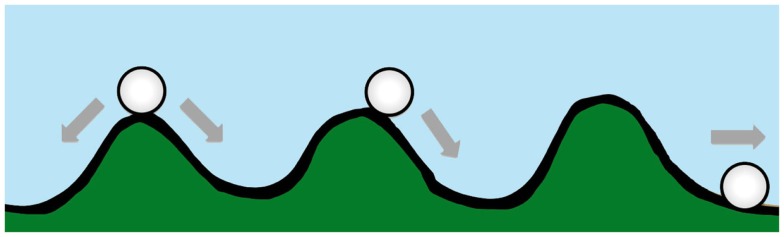
**A diagram conceptualizing Waddington’s epigenetic landscape**. Drug resistance is at an energy maxima (peak), which tends to arrive spontaneously at an energy minima (trough).

Waddington originally proposed the phrase “epigenetic landscape” as a metaphor to describe the process of biological development (34, 35). Huang (36) and other computational biologists have adopted the Waddington metaphor to represent movement on a “rugged” energy landscape composed of high energy crests and low energy troughs. The kinetic consequence of the rugged energy landscape, according to Huang, is that “cells placed on top of a mountain top or at a ‘watershed’ in the epigenetic landscape will roll down into just the few distinct valleys accessible to them, driving the spontaneous separation into discrete fates” (37) (Figure 1).

If the energy landscape metaphor is applied to the chemoresistant phenotype in oncology, and the crest of the hill is a drug-resistant state, which is energetically unfavorable, requiring ATP-consuming transcription and translation of proteins essential for resistance and/or xenobiotic elimination, then in the absence of treatment the cancer cell, like a ball rolling downhill, may spontaneously proceed to the “low energy state” (37) associated with drug sensitivity.

For this reason, the introduction of treatment holidays, during which treatment is suspended for defined periods, may induce reversion to the lowest energy or wild-type baseline drug-sensitive state. Anecdotal cases of resensitization to epidermal growth factor receptor (EGFR) tyrosine kinase inhibitors (TKIs) have been reported in non-small cell lung cancer (NSCLC) patients (38, 39) and genitourinary cancers after temporary treatment interruption “with reported clinical benefits and good tolerability when sequencing back to first-line therapy” (40). Re-treatment responses have also been documented for standard cytotoxic agents (41), which suggest that drug resistance is inherently unstable or metastable, and fades away after selection pressures are removed. Sharma et al. and Knoechel et al. implicate the presence of a subpopulation of drug-tolerant “persister cells,” transiently refractory to killing, that stochastically develop resistance through epigenetic mechanisms, and in particular, chromatin modification, as a kind of “bet-hedging strategy” to generate phenotypic diversity and thereby maximize survival in an uncertain, fluctuating environment without the need to activate energetically intensive stress response pathways; consequently, similar to bacterial cells, when the treatment pressure disappears, so does resistance, which is facultative and reversible because it is epigenetically rather than genetically acquired. This differentiates persister cells, which arise from differential gene expression, from resistant mutants that exhibit stable, irreversible, and heritable drug insensitivity. (42, 43) However, these “passive” resensitization events, which may correlate with the disappearance of the persistence state during a treatment holiday, are welcome but one-off exceptions to the general rule in oncology that chemoresistance is inviolate.

Another “narrow” one-time exemption to the no resensitization rule is the 2004 pivotal BOND (Bowel Oncology and Cetuximab ANtiboDy) trial that served as the basis of FDA (US Food and Drug Administration) approval for Cetuximab (Erbitux^®^), the anti-EGFR monoclonal antibody. In this trial, where metastatic colorectal cancer (mCRC) patients previously treated with irinotecan-based regimens were randomized to cetuximab plus irinotecan or cetuximab alone, the overall response rate (RR) and median overall survival (OS) for the combination arm of cetuximab + irinotecan were 23% and 8.6 months, respectively, vs. 11% and 6.9 months for cetuximab alone, respectively, which provided support for resensitization (44).

These cetuximab resensitization results lack generalizability to a wider population of irinotecan-refractory cancers, since one of the underlying mechanisms of irinotecan resistance is EGFR upregulation (45). As evidence for the stability of this resistance that emerges during therapy, Wadlow et al. published a phase II trial of 20 patients with cetuximab-refractory mCRC treated subsequently with human anti-EGFR monoclonal antibody, panitumumab (Vectibix^®^), where no responses were observed (46).

However, a general rule of inversion does occur in the context of epigenetic priming, which potentially leads to resensitization across multiple tumor types, discussed below.

## Metronomic Dosing

Hanahan, Bergers, and Bergsland described metronomic chemotherapy thusly:*“Less is more, regularly”* (47). In contrast to supratherapeutic MTD bolus chemotherapy, metronomic administration involves regularly timed (like the beat of a metronome) low-dose conventional chemotherapy to avoid both myelosuppression and drug resistance (48). If the drug-resistant state imposes an energetic burden, making resistant cells inferior competitors to their drug-sensitive counterparts, as Gatenby has claimed, then ultra-low doses of chemotherapy may delay but not prevent (49) the onset of treatment resistance. Nevertheless, Hanfeldt et al. (50) have described repeated “resensitization” to chemotherapy after a transient refractory phase with metronomic dosing, which appears to support the “less is more, regularly” slogan.

## Interrupted Dosing

In routine clinical practice, cancer patients are exposed to sequential and multiple lines of *continuous* systemic therapy, with a switch to the next line mandated by objective evidence of radiologic/symptomatic progression or intolerable toxicity, and only rare instances of rechallenge with the same agent. However, the frequency of drug holidays, where treatment is temporarily discontinued, in an attempt to reduce chronic cumulative toxicities and restore partial drug sensitivity, suggests that intermittent administration may be a preferable treatment strategy to continuous dosing.

Three clinical trials in mCRC, OPTIMOX-1, COIN, and AIO KRK 0207 have investigated whether it is safe to introduce chemotherapy-free periods without impacting OS. Each of these trials is briefly discussed below from the perspective of resensitization.

The OPTIMOX-1 trial, intended to address cumulative neurotoxicity with oxaliplatin-based therapy, demonstrated that the interrupted “stop and go” administration of FOLFOX4 treatment for 6 cycles followed by maintenance 5-FU/LV only, with no oxaliplatin for 12 cycles, and reintroduction FOLFOX4 for 6 cycles is as effective as continuous FOLFOX7 until disease progression or toxicity (51) and results in less neurotoxicity. However, resensitization was not demonstrated.

The Phase III COIN trial compared continuous oxaliplatin and fluoropyrimidine vs. 3 months of oxaliplatin and fluoropyrimidine followed by a preplanned chemotherapy holiday until disease progression and then treatment reinduction. Resensitization was not demonstrated. Survival of about 6 weeks was noted in the intermittent arm, but this still did not support resensitization.

AIO KRK 0207 investigated the effect of a 24-week standard induction with FOLFOX + bevacizumab, no continuation of therapy or continuation with bevacizumab alone, followed by reinduction after progression. This study also did not demonstrate resensitization, since time to failure of strategy, a composite measure of progression-free survival, was worse in the no treatment arm.

The absence of resensitization on the intermittent or no treatment arms may be related to protocol non-adherence since in all three trials the reinduction rate was low, possibly due to the toxicity of treatment (52, 53).

## DNA Methyltransferase Inhibitors

Similar to the “less is more” principle that characterizes metronomic dosing (54). the azanucleotides, AZA, and DAC are more effective at lower “epigenetically targeted” concentrations due to inhibition of DNMTs (55) and global hypomethylation, which leads to re-expression of silenced tumor suppressor genes.

**Figure 2:**
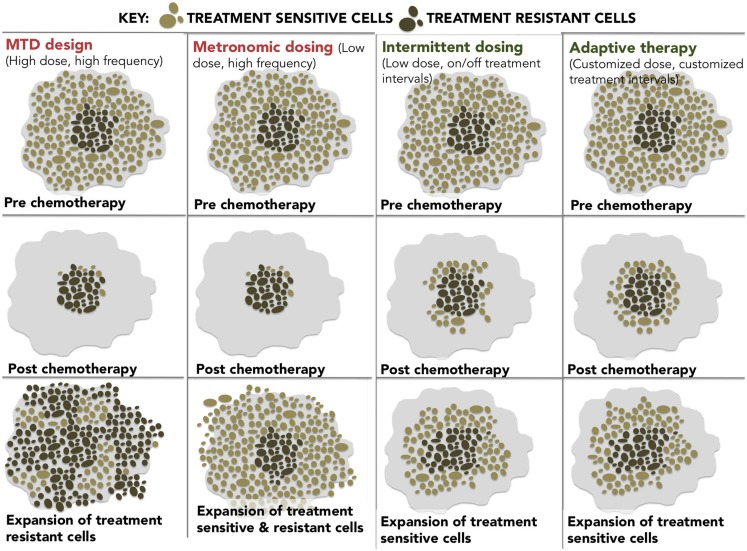
**A diagram showing four different dosing strategies**. (1) MTD dosing – maximal doses with maximal resistance. (2) Metronomic dosing – less is more regularly. (3) Intermittent dosing –start and stop dosing theoretically leads to less resistance than continuous dosing. (4) Adaptive therapy – dosing level and frequency adapted for tumor stability.

At these epigenetically targeted doses, *systematic* observations of resensitization to previously failed therapies have been reported most notably in platinum-refractory ovarian cancer. The word systematic is italicized to highlight the difference between active and spontaneous resensitization events. In the former, hypomethylating agents elicit a predictable and purposeful pattern of altered gene expression, while spontaneous resensitization (e.g., from pulsed or intermittent treatment schedules) occurs inconsistently and unpredictably. Reversion to drug sensitivity may happen eventually; however, due to randomness, it is evidently impossible to predict when (or if) that event will occur, which renders spontaneous resensitization of an unreliable strategy. The different examples of non-epigenetic resensitization are represented below (Figure 2).

## Clinical Trials that Feature Epi-Resensitization

Ovarian cancer, hepatocellular carcinoma (HCC), colorectal cancer, NSCLC, and cholangiocarcinoma are some of the tumor types where an episensitization strategy has already been explored or is currently under investigation.

**Figure 3:**
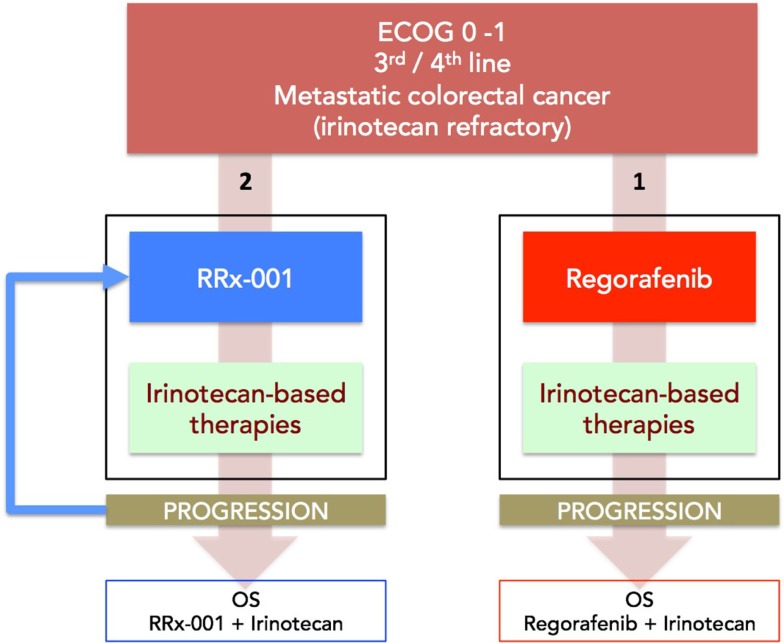
**A diagram depicting the ROCKET trial design**. Patients with mCRC are randomized to RRx-001 or regorafenib. On progression, both arms are rechallenged with irinotecan-based therapies, if clinically appropriate. The primary endpoint is OS. Another endpoint is PFS measured as the sum of PFS (RRx-001 or regorafenib) + PFS (irinotecan therapies).

**Figure 4:**
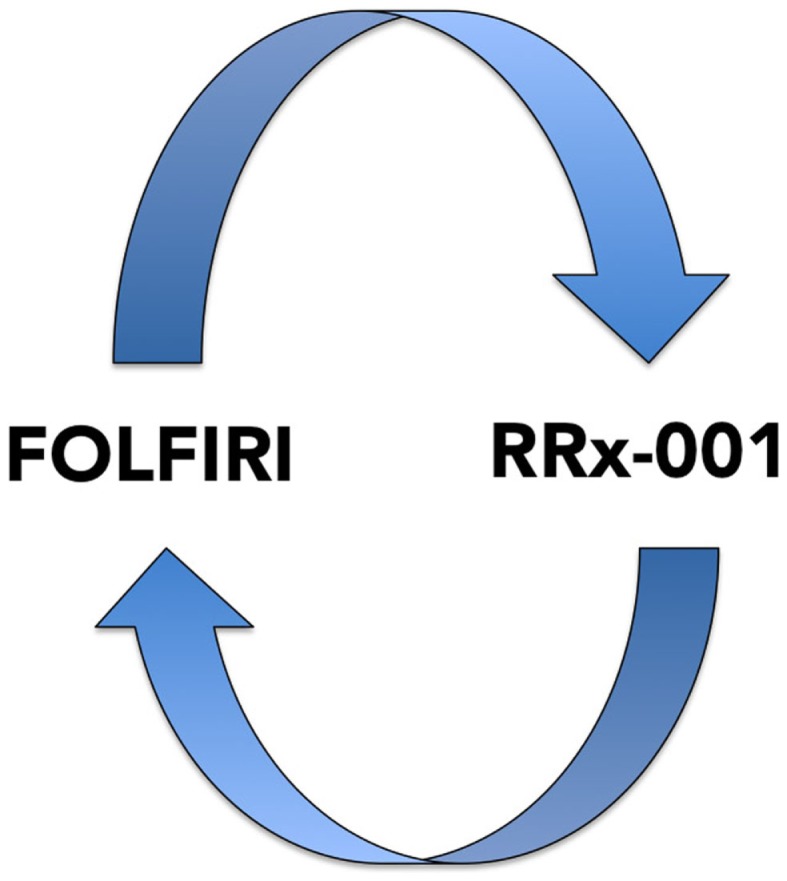
**A diagram depicting the RRx-001 resensitization loop**. In the ROCKET trial, RRx-001 patients are shuttled between RRx-001 and FOLFIRI in an iterative retreatment loop.

(1) Ovarian cancer
  Standard first-line therapy for advanced ovarian cancer consists of a combination of a platinum and a taxane usually carboplatin and paclitaxel administered intravenously every 3 weeks for six cycles (56). Nevertheless, while most tumors are initially responsive, patients inevitably develop resistance to platinum and all other therapies, at which point their 5-year survival rate drops to only 20% (57) because recurrent disease is almost always incurable. Decision-making for second-line treatment is based on a three category clinical classification, platinum-refractory, platinum-resistant, and platinum-responsive, defined by the time interval of relapse after the end of first-line platinum-containing chemotherapy regimen (58): platinum-refractory tumors progress during platinum-based therapy and have the worst prognosis; platinum-resistant disease recurs <6 months after platinum-based therapy; platinum-sensitive disease recurs more than 6 months from the completion of the initial platinum-based therapy (59). With the exception of platinum-sensitive disease, which may benefit from retreatment with either cisplatin or carboplatin, secondary response to platinum-based agents for resistant/refractory patients (60) is extremely unlikely.
  (a) Phase II clinical trial of DAC and carboplatin in platinum-refractory/resistant ovarian cancer.
    Matei et al. (61) tested the activity of AZA and carboplatin in 17 patients with heavily pretreated and platinum-resistant ovarian cancer in a phase II clinical trial. RR and progression free survival were 35% and 10.2 months, respectively, indicative of epi-resensitization and clinical benefit since objective responses are not only uniformly short-lived but <10% in this patient population (62).
  (b) Phase I/II study of 5-azacytidine and carboplatin platinum-refractory/resistant ovarian cancer.
    In this similar trial, Fu et al. (63) administered AZA 75 mg/m^2^ subcutaneously daily for 5 days to reverse resistance to carboplatin in patients with platinum-resistant or refractory ovarian cancer. The fact that the overall RR was 13.8% (4 of 29 patients) with 1 patient achieving a complete response and 3 patients achieving a partial response (PR); the disease control rate [PR plus stable disease (SD)] was 45% (13 of 29 evaluable patients) and the median OS was 14 months, is also indicative of epi-resensitization since in this subset of patients the probability of benefit with carboplatin is unlikely.
(2) Advanced HCC.
  The multikinase inhibitor, sorafenib (64), is the first-line standard of care in HCC patients, however, RRs are low, resistance to therapy inevitably develops, and no approved therapies are available in the second-line setting. Resminostat is an oral pan-HDAC inhibitor.
  (a) Phase II SHELTER study of resminostat + sorafenib vs. resminostat alone in second-line HCC after sorafenib progression in first-line treatment.
    The OS is reportedly 8.1 months in the resminostat + sorafenib arm vs. 4.1 months for resminostat alone (65). The expected OS in second-line HCC, in other words, after patients progress on sorafenib is 5.2 months, resulting in a modest survival benefit of about 3 months in the combination sorafenib/resminostat epi-resensitization arm.
(3) mCRC.
  Unlike the fairly rigid dependence on serial lines of treatment in the majority of tumor types, a flexible “continuum of care” (66) model characterizes the management of metastatic colon cancer. Continuum of care involves strategic pairing of approved chemotherapy agents, including 5-FU, irinotecan, bevacizumab, capecitabine, panitumumab, cetuximab, and regorafenib in any order (67); however, rechallenge with formerly failed therapies is typically not attempted since the risks likely outweigh the potential benefits. The recently approved agent, regorafenib, is recommended for third-line therapy in patients pretreated with fluoropyrimidines, oxaliplatin, irinotecan, bevacizumab, or anti-EGFR antibodies.
  The epigenetics of colorectal cancer has been reviewed extensively and preclinical studies suggest that epigenetic therapies like vorinostat may reverse chemoresistance in colorectal tumors (68).
  In addition, preliminary clinical evidence demonstrates that the epigenetic agent, RRx-001, a systemically non-toxic intravenous agent, sourced from the aerospace industry, reverses chemoresistance to irinotecan in a randomized Phase II mCRC trial vs. regorafenib with the acronym ROCKET for RRx-001 in COlon Cancer TaKen To Extend Time. This trial systematically explores a switch-at-progression strategy of RRx-001 priming followed by irinotecan, RRx-001, then irinotecan, etc.
  (a) Phase II (ROCKET) study of weekly RRx-001 randomized against current third treatment, regorafenib, and followed by irinotecan-based therapies on progression (Figure 3).
    This recently initiated Phase II two-part clinical trial consists of a priming phase and a successive cytotoxic chemotherapy phase. Previously treated irinotecan-refractory third/fourth-line colorectal cancer patients are randomized to RRx-001 or regorafenib administered until progression, intolerable toxicity, or patient choice. Per protocol irinotecan-based therapy is reintroduced on both arms, if clinically appropriate. In addition, the RRx-001 patients, if appropriate, are allowed to continue on trial in a repetitive retreatment loop, a circular dichotomization that alternates between RRx-001 and irinotecan-based therapies (Figure 4).
    Epi-resensitization is defined by any of the following (1) a drop in carcinoembryonic antigen (CEA) levels; (2) clinical and/or radiologic stability; (3) prolonged clinical benefit on therapy leading to increase OS. The primary endpoint is OS.
    To date, six out of eight RRx-001 patients have been successfully rechallenged with irinotecan-based therapies after completion of the first priming phase of the study. These six patients met criteria for resensitization with one or more of the following: a (1) decline in the tumor-associated marker carcinoembryonic antigen (CEA); (2) improvement in clinical performance status; and/or (3) PR/SD on positron emission tomography (PET) or computer tomography (CT). One of the patients demonstrated a metabolic PR on PET and SD on CT. In contrast to the RRx-001-treated arm, the regorafenib patients all clinically deteriorated and have been unable to start any subsequent chemotherapy. The primary hypothesis is that the successful reintroduction of previously effective therapies with subsequent regression or prolonged stabilization of disease will improve long-term survival rates compared to the regorafenib control arm.
    These early examples suggest that RRx-001 epigenetically primes tumors to re-respond to irinotecan-based therapies. The prolonged nature of resensitization to irinotecan-based therapies are akin to those of treatment-naïve mCRC patients, who have never been previously exposed to chemotherapy, since the median PFS in second-line metastatic colorectal therapy is 4.5 months (69).
  (b) Phase I Study of SGI-110 combined with irinotecan followed by a randomized phase II study of SGI-110 combined with irinotecan vs. regorafenib in previously treated mCRC patients.
    In this recently initiated two-part combination Phase I clinical trial, the combination of the second generation DNMT inhibitor, SGI-110, and irinotecan is compared to the standard of care regorafenib in mCRC. No data are available.
(4) NSCLC.
  In patients with advanced or metastatic NSCLC, the median survival is 10–12 months. The current first-line chemotherapy standard is one of a number of platinum-based doublets followed in second line by treatment with the chemotherapy agents docetaxel and pemetrexed, or treatment with an oral EGFR antagonist, erlotinib. These patients have limited treatment options (and poor outcomes) after progression in second-line. Retrospective analysis of third-line chemotherapy shows RRs of only 2% and median survival of 4 months (70).
  (a) Phase I/II clinical trial of combined epigenetic therapy with AZA and entinostat in extensively pretreated recurrent metastatic NSCLC.
    This trial demonstrates tumor preconditioning or priming prior to subsequent therapies, which is consistent with episensitization. Treatment with low-dose AZA and entinostat by Juergens et al. (71) achieved a favorable objective RR and survival benefits (>1 year in approximately 20% of the patients and a median OS of 6.4 months), which exceeded historical controls (48% expected survival after 6 months). The favorable survival was attributed to an “unusually robust response to subsequent cytotoxic therapies with which the majority of patients were treated” (9). The subsequent therapies in the NSCLC trial included pemetrexed, docetaxel, erlotinib, anti-programed cell death protein (PD-1) monoclonal antibodies, gemcitabine, irinotecan/bevacizumab, and cisplatin, suggesting that the combination of AZA and entinostat reversed the multimodality resistance phenotype, thereby enhancing susceptibility to a variety of subsequent chemotherapeutic agents.
  (b) Pilot Three-Arm Study (TRIPLE THREAT) of RRx-001 administered in small cell lung cancer, NSCLC, and extrapulmonary neuroendocrine tumors prior to re-administration of platinum-based doublet regimens – this trial is scheduled to start recruitment in Q1 2015.
(5) Cholangiocarcinoma
  Cholangiocarcinoma is a rare, heterogeneous group of tumors with an incidence that ranges from 1 to 3 per 100,000 in the United States (72). The majority of patients who develop cholangiocarcinoma are incurable; palliative gemcitabine plus platinum combination chemotherapy is standard practice (73) as first-line treatment while the benefits and feasibility of second-line salvage chemotherapy are unclear and under investigation.
  (a) Phase II (EPIC) trial of weekly RRx-001 administered for 6 weeks in second-line therapy prior to re-administration of gemcitabine/cisplatin – this trial is scheduled to start recruitment in 2015.

## Discussion and Future Perspective

**Figure 5:**
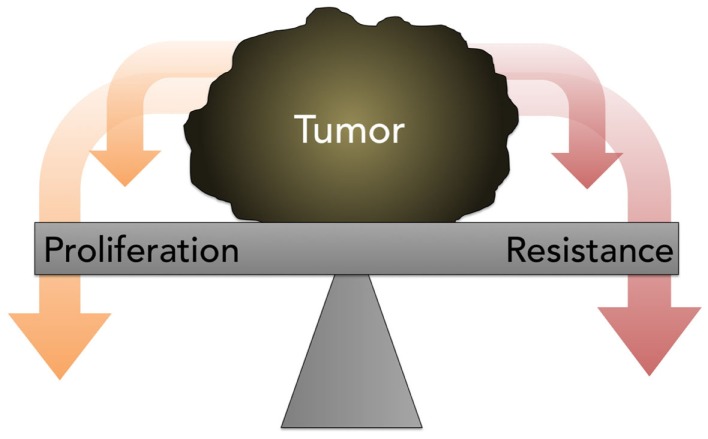
**A diagram depicting the concept of proliferation- resistance trade-offs**. Tumors allocate finite resources toward proliferation or resistance, depending on the presence or absence of specific stresses. This process is referred to as the proliferation-resistance trade-off.

The traditional assumption in oncology that resistance is inevitable, stable, and homogeneous, eventually leading to tumor progression and death, with small chance for alleviation or reprieve, generally prevents a retrial of previously effective chemotherapy due to the risk of toxicity without benefit. Contrary to this prevailing view, the work of Huang and Gatenby, in particular, cited earlier, demonstrates that acquired resistance, energetically expensive to produce and maintain, is potentially a context-dependent and -reversible process. As explained by Gatenby, one-size-fits-all MTD-based treatment is fuel for the evolution and emergence of resistance against multiple chemotherapies (74). Because cancer cells possess a finite pool of resources, due to a decreased efficiency of energy metabolism via glycolysis and thus ATP production, the possession of these defense mechanisms is a metabolically costly trade-off that compromises proliferation (Figure 5); this negative impact on proliferation is only supportable when the associated benefits outweigh the costs. It is hypothesized that when the external selection pressure is removed, the tumor will revert to baseline and adopt, on balance, a drug-sensitive phenotype. However, it is unpredictable if, when, and how quickly the cell will overcome phenotypic inertia. Moreover, the very act of treatment elicits a Catch-22 of resistance even after a chemotherapy-free interval.

The apparent solution to this paradoxical situation is epigenetic modulation, because it globally modulates gene expression, which disrupts the specialized fit between the cancer cell and its microenvironment, forcing the tumor to recalibrate and reset at the expense of *de novo* and acquired resistance. While reversal of resistance may occur spontaneously during treatment interruption, resensitization is more reliably elicited with epigenetic agents, which justifies the use of the term epi-resensitization.

In conclusion, episensitization has the potential to increase the expectancy of survival from mere months to multiple years, as resensitized patients iteratively loop between epigenetic agents and formerly refractory treatments in consecutive cycles. The goal, 1 day, is to bend or hopefully even turn back the one-way arrow of time and transform cancer from a terminal to a chronic disease.

## Conflict of Interest Statement

The authors declare that the research was conducted in the absence of any commercial or financial relationships that could be construed as a potential conflict of interest.

## Footnotes

^1^http://en.wiktionary.org/wiki/resensitization. Accessed December 2014

## Abbreviations

ATP, adenosine triphosphate; AZA, azacitidine; BOND, bowel oncology and cetuximab antibody; CEA, carcinoembryonic antigen; CT, computer tomography; DAC, decitabine; DNMT, DNA methyltransferase; EGFR, epidermal growth factor receptor; FDA, US food and drug Administration; GI, gastrointestinal; GU, genitourinary; HCC, hepatocellular carcinoma; HDAC, histone deacetylase; MTD, maximally tolerated dose; mCRC, metastatic colorectal cancer; NSCLC, non-small cell lung cancer; OS, overall survival; PET, positron emission tomography; PR, partial response; ROCKET, RRx-001 in colon cancer taken to extend time; SD, stable disease; TKIs, tyrosine kinase inhibitors; VEGF, vascular endothelial growth factor.
